# Preclinical Orthostatic Abnormalities May Predict Early Increase in Vascular Stiffness in Different Age Groups: A Pilot Study

**DOI:** 10.3390/diagnostics13203243

**Published:** 2023-10-18

**Authors:** Victor N. Dorogovtsev, Dmitry S. Yankevich, Andrey E. Gaydashev, Andrey V. Martyushev-Poklad, Julia A. Podolskaya, Ilya V. Borisov, Andrey V. Grechko

**Affiliations:** 1 Federal Research and Clinical Center of Intensive Care Medicine and Rehabilitology, 107031 Moscow, Russia; dyankevich@fnkcrr.ru (D.S.Y.); amp@fnkcrr.ru (A.V.M.-P.); upodolskaya@fnkcrr.ru (J.A.P.); noo@fnkcrr.ru (A.V.G.); 2 Problem Scientific Research Laboratory, Smolensk State Medical University, 214019 Smolensk, Russia; pnil-kardio@yandex.ru

**Keywords:** head-up tilt test, arterial hypertension, blood pressure, preclinical orthostatic hypotension, preclinical orthostatic hypertension, pulse wave velocity, vascular stiffness, vascular aging

## Abstract

Clinical orthostatic hypotension (OH) and hypertension (OHT) are risk factors for arterial hypertension (AH) and cardiovascular diseases (CVD) and are associated with increased vascular stiffness. Preclinical OH and OHT are poorly understood. The main objective was to investigate preclinical orthostatic abnormalities and their association with increased vascular stiffness in different age groups of adults. A specially designed head-up tilt test standardized for hydrostatic column height was used to detect them. Three age groups of clinically healthy subjects were examined. In the group of young adults up to 30 years old, a significant predominance of orthostatic normotension (ONT) and an insignificant number of subjects with preclinical OH and OHT were found. In the age group over 45 years, compared to the group under 30 years, there was a twofold decrease in the proportion of individuals with ONT and a significant increase with preclinical OH and OHT. In all age groups, there was a significant orthostatic increase in vascular stiffness (as measured by the brachial–ankle pulse wave velocity (baPWV), which was recovered to the baseline level when returning to the supine position. Overall, subjects with preclinical OH and OHT had significantly higher baPWV values compared to those with ONT (*p* = 0.001 and *p* = 0.002, respectively), with all subjects having vascular stiffness values within normal age-related values.

## 1. Introduction

Clinical orthostatic hypotension (OH) and clinical orthostatic hypertension (OHT) increase the risk of arterial hypertension (AH) and cardiovascular diseases (CVD) [1,2,3]. In the older population, a significant increase in vascular stiffness accompanies OH and OHT [4,5,6]. Such a connection is of great practical relevance because increased vascular stiffness is one of the most important risk factors for arterial hypertension (AH) and CVD [7,8,9,10,11]. Age-related changes in blood vessels are manifested by structural changes in the vascular wall, leading to an increase in its stiffness [12,13]. The state of vasculature determines the biological age of an individual, which can differ significantly from chronological age [14].

Many factors are known to accelerate vascular aging, such as increased blood pressure, atherosclerosis, smoking, increased sugar consumption, alcohol consumption [15], diabetes (DM) [16], chronic kidney disease [17], metabolic syndrome [18], and many others. Some pathologic processes occurring in the blood vessels can also significantly accelerate the process of stiffening, such as endothelial disorders [19], chronic Inflammation [20], oxidative stress [21], etc. There is a clear need to study the early stages of processes that accelerate vascular aging for their early diagnosis, control, and correction. 

Gravity has a strong influence on the cardiovascular system (CVS) throughout human and animal life. Evolutionary aspects of physiological, ontogenetic, and clinical factors and their effects on humans and animals are actively studied [22,23]. It has been hypothesized that essential hypertension may be a natural adaptation to gravitational stress [22,24]. A special feature of our approach was to consider preclinical asymptomatic orthostatic abnormalities as impaired orthostatic circulatory regulation, contributing to the development of AH and increased arterial stiffness.

We consider it promising to study orthostatic shifts in the cardiovascular system occurring during the change of body position. On the one hand, these changes are determined by the influence of gravity and, on the other hand, by the activation of adaptive systems. Orthostatic normotension (ONT) is a sign of adequate function of adaptive systems, in contrast to OH and OHT, which are accompanied by higher CVD risk and by increased vascular stiffness (see above). Clinical orthostatic disorders due to primary autonomic nervous system diseases or secondary disorders are not considered in our work. 

In contrast to clinical orthostatic abnormalities, the association of preclinical abnormalities with vascular stiffening remains poorly understood [25,26]. Nevertheless, it has been shown that orthostatic abnormalities corresponding to preclinical OHT significantly increase the risk of AH in a young population under 30 years of age [27], and preclinical OH increases the risk of AH in a population over 45 years of age [28]. In the latter study, the risk of AH was rising in proportion to the increase in stiffness as measured by intima-media thickness in preclinical OH, with stiffness reaching its peak with the development of clinical OH. These data suggest that clinical orthostatic disorders may be the result of the evolution of preclinical ones. Specifically for the detection of preclinical orthostatic abnormalities, we developed a new passive orthostatic test (HUTT) standardized by the height of the hydrostatic column, providing a standard gravity load for all subjects regardless of their height [29].

We hypothesized that the phenotypes of preclinical orthostatic abnormalities may be different in different age groups, but all may be associated with increased vascular stiffness compared with ONT.

The main objective was to investigate preclinical orthostatic abnormalities and their association with increased vascular stiffness in different age groups of adults with the use of HUTT standardized by the height of the hydrostatic column.

## 2. Methods

### 2.1. Participants

Inclusion criteria: adults undergoing annual health check-ups, without an exacerbation of an existing illness, non-smokers, not taking coffee or alcohol 24 h prior to experiment. Individuals with an established diagnosis of chronic disease were included if their clinical and laboratory parameters corresponded to the age norm.

Exclusion criteria: cardiac arrhythmias, acute heart disease, history of the CVD, peripheral arterial blood flow disorders, history of orthostatic intolerance, peripheral edema, signs of thrombophlebitis or complicated varicose veins, body mass index over 30 kg/m^2^.

Measurements were carried out 2 h after a light breakfast, between 8:00 and 11:30 a.m., in a laboratory with ambient temperature between 24 and 25 °C, 50–55% humidity, and minimal noise level. Women of reproductive age were investigated outside of menstruation period.

### 2.2. Head-Up Tilt Test (HUTT) Procedure

The HUTT was performed using an electrically operated tilt table and according to the Luanda protocol, which consisted of 3 phases: supine position for 10 min; HUTT position for 10 min; return to supine position for 10 min. This protocol included the setting of an individual tilt angle to establish the standard height of 133 cm for the hydrostatic columns of all participants, irrespective of their height [29].

### 2.3. Hemodynamic Measurements

Two BP measurements from the 6th to 10th minute of each of the three positions on brachial and ankle arteries and brachial–ankle pulse wave velocity (baPWV) were performed using multichannel volumetric sphygmography method on an ABI-System 100 PWV (BOSO, Berlin, Germany). Brachial systolic blood pressure (SBP), diastolic blood pressure (DBP), pulse blood pressure (PBP), heart rate (HR), and baPWV were measured twice and averaged for each of the three positions. This device performed a computerized calculation of the carotid–femoral pulse wave velocity (cfPWV), and the accuracy of the calculation of this parameter has been validated [30]. We used this indicator only to analyze the baseline data in the horizontal position of the subjects (I supine). 

### 2.4. Identifying Preclinical Orthostatic Abnormalities in Different Age Groups

The difference in SBP values during HUTT and in the horizontal position (ΔSBP) allows the detection of preclinical orthostatic abnormalities. Preclinical OH corresponds to ΔSBP values −20 mmHg < ΔSBP < −5 mmHg, preclinical OHT was diagnosed at values +5 mmHg < ΔSBP < +20 mmHg, orthostatic normotension (ONT)—ΔSBP between ±5 mmHg [26,29]. Orthostatic intolerance: clinical OH—from −20 mmHg and below, and clinical OHT from +20 and above were not evaluated in our study as were included in the exclusion criteria [31,32].

### 2.5. Statistical Analysis

Statistical analysis was carried out using STATISTICA 10 software (TIBCO Software, Palo Alto, CA, USA). Nominal data were described with absolute values. Quantitative data with a normal distribution were combined into a variation series, in which the arithmetic mean (M) and standard deviations (SD) were calculated. In the absence of a normal distribution, quantitative data were presented as median and quartiles (25–75% margins of the interquartile range). The Kolmogorov–Smirnov test was used to verify the nature of the distribution. When comparing the mean values in normally distributed populations of quantitative data, Student’s *t*-test was calculated. Mann–Whitney U-test was used to compare two independent groups when there was no evidence of normal distribution. The Kruskal–Wallis test was used when comparing several samples of quantitative data with a distribution other than normal. Wilcoxon’s W test was used to analyze the statistical significance of differences in quantitative characteristics for two dependent samples. Differences were considered statistically significant at *p* < 0.05.

## 3. Results

In total, 120 subjects 20–70 years old were included in this study, all divided into 3 age groups: Group 1—20–30 years old, Group 2—31–45 years old, and Group 3—over 45 years old. Due to the small sample size, to study the association of preclinical orthostatic abnormalities with increased stiffness, we combined data from all three age groups and excluded subjects with diagnoses of AH (18 subjects).

Descriptive and comparative characteristics of the subjects in three age groups are presented in Table 1.

The health status of all subjects was checked during annual health check-ups. The results are presented in Table 2.

The health status of the subjects in different age groups is presented in Table 2. In Groups 1, 2, and 3, correspondingly, 92.5%, 67.5%, and 55% of individuals had no health problems according to the ambulatory check-up. None of the individuals with diagnosed diseases experienced an ongoing aggravation, and all clinical and biochemical parameters were within the limits of age norms. The most common diseases detected in Groups 2 and 3 were arterial hypertension, atherosclerosis, and diabetes.

Orthostatic changes in blood pressure (BP) and pulse wave velocity (PWV) in different age groups are shown in Table 3.

The following differences in baseline data were found in the analysis of averages in three age groups: lower HR and DBP values in Group 1 compared with Group 2 (*p* = 0.004) with no significant difference in SBP. The average baseline values of cfPWV were minimal in Group 1, and they increased progressively with age in Group 2 (*p* < 0.006) and Group 3 (*p* = 0.002).

A significant increase in HR during HUTT was detected in Groups 2 and 3 (*p* < 0.001) (Table 3). Intra-group orthostatic changes consisted of a slight increase in SBP in Group 1 and Group 2 (*p* = 0.03), and significant increases in DBP and HR (*p* < 0.01) compared to the horizontal position. During HUTT standardized by hydrostatic column height, baPWV increased significantly in all groups (all inter-group differences *p* = 0.001).

Returning to the supine position caused all parameters to recover to baseline values (*p_I–III_* > 0.05), except Group 2 which featured higher HR (*p* = 0.05) in the HUTT position. In Group 3 PWV during recovery was lower than at the baseline (*p* < 0.001).

**Figure 1:**
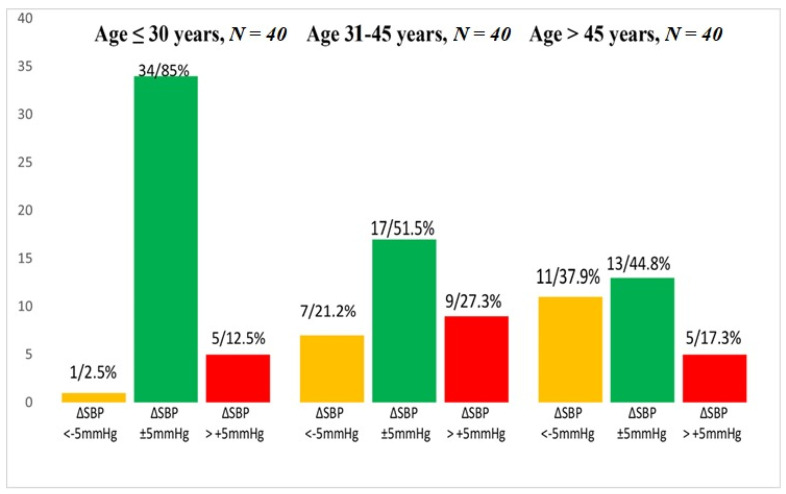
Number of subjects in 3 age groups with and without preclinical orthostatic disturbances. Note: Y-axis—Number of subjects, yellow column—preclinical orthostatic hypotension (preclinical OH) (ΔSBP < −5 mmHg), green column—orthostatic normotension (ONT) (ΔSBP ± 5 mmHg), red column—preclinical orthostatic hypertension (preclinical OHT) (ΔSBP > +5 mmHg).

Preclinical orthostatic abnormalities in different age groups are shown in Figure 1.

As expected, the younger Group 1 featured the largest proportion of preclinical ONT (85%), with preclinical OH, a potential predictor of AH, in 12.5% and preclinical OH in only 1 subject in this group (2.5%). In Group 2, the prevalence of orthostatic normotension was lower (51.5% vs. 85%) due to the higher prevalence of preclinical OHT and OH. In Group 3 the prevalence of ONT went further down (to 44.8%) due to much more frequent preclinical OH, while preclinical OHT was less prevalent than in Group 2. The small sample size of the groups did not allow performing a comparative BP and PWV analysis within the age groups.

The prevalence of preclinical orthostatic abnormalities was associated with higher vascular stiffness in the studied adults.

To address the main question of this study, we excluded all subjects diagnosed with AH (18 subjects). The remaining subjects (*N* = 102) were divided into three groups according to the presence or absence of preclinical orthostatic abnormalities (Table 4).

Baseline HR did not differ in all three groups (*p* > 0.05), significantly higher SBP and DBP values were observed in Group 1 with preclinical OH, and the lowest values for these parameters were observed in Group 3 and OHT group. Significant differences were found in baseline PWV (Table 4). Subjects with ONT (Group 2) showed minimal values of baPWV and cfPWV compared to preclinical orthostatic hypotension (Group 1) (*p* < 0.01) and hypertension (Group 3) (*p* < 0.01). At the same time, no significant difference in baseline PWV was found in subjects with preclinical orthostatic abnormalities (between Groups 1 and 3) (*p* > 0.05).

During HUTT of the standardized hydrostatic column height, there was no significant increase in HR in all groups (*p* < 0.001). In Group 1, SBP decreased significantly (*p* < 0.001); Group 3 featured a significant increase in SBP (*p* < 0.001); Groups 2 and 3 demonstrated a significant increase in DBP (*p_I–II_* < 0.001) (Table 4). HUTT PWV significantly increased in all groups compared to the horizontal position (in all groups *p_I–II_* < 0.001). During HUTT, the highest SBP was detected in Group 3 compared to Group 1 (*p* < 0.01) and Group 2 (*p* = 0.004). The differences between groups in PWV detected in the horizontal position were also maintained in HUTT: minimal values were observed in Group 2 with ONT and higher values in subjects with preclinical OH and OHT. Moreover, there was no difference in PWV values between Group 1 with preclinical OH and Group 3 with preclinical OHT (*p* > 0.05).

On return to the horizontal position, almost all indicators returned to baseline values in all groups.

## 4. Discussion

Baseline blood pressure (BP) values in all age groups were within age-related norms (Table 3). Inter-group differences in baseline data consisted of lower HR and BP values in the young adult group compared to the older groups. Group analysis of orthostatic BP and HR changes revealed typical changes: insignificant changes in SBP, and significant increases in DBP and HR during HUTT compared to the horizontal position, which were consistent with those reported in the literature [33,34]. After returning to the horizontal position, almost all indices returned to the initial values. Averaged hemodynamic indices obtained in group analysis in a population of clinically healthy people are of limited value, as they do not allow identifying personalized data that carry important information about the presence or absence of preclinical orthostatic abnormalities.

The baseline cfPWV and baPWV values were within the age norm criteria in all age groups (Table 3); the lowest values were in the group under 30 years of age, and the highest in subjects over 45 years of age, which was in conformity with the literature [12,35]. Our study revealed a significant increase in baPWV in all three age groups when performing HUTT creating a standard gravity load: in Group 1 from 8.9 [8.3; 9.5] to 12. 9 [11.9; 14.2] (*p* < 0.001), in Group 2 from 10.1 [9.7; 11] to 13.8 [13; 14.5] (*p* < 0.001) and in Group 3 from 11.3 [10.7; 11.9] to 14.3 [13.3; 15.4] (*p* < 0.001). The increase in median PWV at the tilted versus supine position was 4.0, 3.7, and 3.0 m/s in Age Groups 1, 2, and 3, respectively. In a similar study, when cfPWV was measured during CUTT 0°–30°–60° in a population of 45 ± 18 years old, cfPWV was 8, 9.1, and 9.5 m/s, respectively, i.e., the increase in cfPWV was only 1.1 and 1.5 m/s [36]. The larger increase in baPWV compared to cfPWV is probably due to the different locations of the PWV measurement. In the first case, the measurement is performed on a section of the vascular system, including muscular arteries, mixed-type arteries, and the aorta, while in the second case, it is performed predominantly only on the aorta.

In our study, the prevalence of ONT, preclinical OH, and OHT in the group of adults under 30 years of age differed from a prototype study [28]: 85% vs. 57.2% for ONT, 2.5% vs. 26.6% for preclinical OH, and 12.5% vs. 16.2% for preclinical OHT. We attribute the difference in results to the peculiarities of the applied protocols: in our study, we assessed orthostatic regulation of hemodynamics using HUTT with standardized hydrostatic column height. In the prototype study with the active standing test, orthostatic changes depended on the peculiarities of orthostatic regulation and individual subjects’ height (hydrostatic column height). We believe that our results may be more accurate.

With aging, the prevalence of ONT was declining, and interestingly, in the group over 45 years of age preclinical OH was more prevalent than preclinical OHT (Figure 1). These findings from the cross-sectional study suggest a possible evolution of preclinical orthostatic deviations, which is expressed as the development of preclinical orthostatic abnormalities in a part of subjects with normal orthostatic regulation (ONT). Presumably, the occurrence of preclinical OHT was lower in Age Group 3 because a large part of individuals of this age had already developed clinical AH and thus were not included in the study.

Unfortunately, it is not possible to make a comparative analysis of the data obtained in these age groups with the data of the prototype study due to the difference in study protocols [28]. However, we want to focus on two important results of the last study, the prototype: (1) the risk of AH was increasing from the ONT boundary toward clinical OH, at which it reached a maximum and (2) the increase in AH risk was accompanied by a progressive increase in vascular stiffness as measured by intima-media thickness. These data from prototype studies allowed us to design and conduct the present study, and most importantly its final phase, which aimed to identify the association of preclinical asymptomatic orthostatic abnormalities with increased arterial stiffness as measured by baPWV.

Due to the small sample size, at this stage of the research, data from all subjects were pooled (except for 18 individuals with AH) and divided into three groups: with preclinical OH, OHT, and with ONT (Table 4). Baseline SBP, DBP, and baPWV were significantly higher in the preclinical OH group compared to the ONT and preclinical OHT groups (*p* < 0.02), while no differences were found for HR (*p* > 0.25). As follows from the study protocol, during HUTT, SBP and DBP went down in preclinical OH (*p* = 0.001), went up in the OHT group (*p* < 0.001), and were stable in the ONT group. HR increased in all groups, but the average changes were significant in only ONT. When returning to the horizontal position, almost all indices reached the initial values (Table 4).

In our study, baPWV values were within normal limits in all groups. At the same time, comparative statistical analysis revealed an association of preclinical OH and OHT with an early increase in vascular stiffness: these groups featured significantly higher baseline values of baPWV compared to ONT (*p* < 0.001). These data were consistent with the results of a prospective prototype study [28]. This fact may be of great importance if we assume the existence of age-related evolution of preclinical orthostatic abnormalities into clinical orthostatic disorders in which vascular stiffness is significantly increased.

Orthostatic increases in arterial stiffness have been shown in studies using standard techniques [37,38]. A similar effect was found in our previous studies and in the present work with the application of HUTT standardized by hydrostatic column height [26,29]. Orthostatic elevation of baPWV may be clinically relevant because it is a proven indicator of the CVD risk when it reaches 15.9 m/s or higher [39]. In another study, baPWV from values of 14.0 m/s and above was shown to be a predictor of CVD [40]. Meta-analysis of cohort studies found a 12% increased risk of CVD with an increase in baPWV of 1 m/s [41]. In our study, during tilt up, subjects with preclinical OH and OHT exceeded the CVD risk boundary (14.0 m/s). The orthostatic increase in PWV was transient and quickly recovered when returning to the initial position on the back, as shown in our study (Table 4).

A special feature of our study was the inclusion of clinically healthy adults who underwent annual health check-ups. Rigorous participant selection aimed to minimize the impact of comorbidities on the results of the present study partly explains the relatively small sample size in this pilot study. We included all subjects in the group analysis. In the main step of identifying the association of preclinical abnormalities with increased vascular stiffness before the development of AH, all individuals with this disease were excluded. All three age groups recruited subjects with normal body weight because obesity is a risk factor for AH [42,43]. The second and third age groups included subjects with various diseases (see Table 2). The criterion for the inclusion of clinically healthy subjects in our study was compliance of clinical, biochemical, and instrumental parameters detected during annual health check-ups with age-related criteria of normality.

We consider the results of two representative prospective studies as prototypes for our pilot study [27,28]. These studies were the first to show the predictive ability of preclinical orthostatic abnormalities in young and old healthy populations and the first to identify the association of such abnormalities in an older population with a more rapid increase in vascular stiffness compared with ONT. In representative prospective studies—prototypes—preclinical orthostatic abnormalities were evaluated in the test of active standing from the initial positions of sitting or lying down [44,45]. This test had been proposed to determine the personalized risk of syncope [46]. Its main feature is the formation of a maximum hydrostatic column height for the tested individual, which is directly related to height. Such a method may be inadequate for detecting preclinical, asymptomatic orthostatic abnormalities, which reflect the state of orthostatic regulation of blood circulation.

The sympathetic baroreflex and vestibulo-sympathetic reflexes are activated to ensure the stability of organ blood flow when moving from a prone to tilt up or upright position [47,48,49]. In addition to the activation of the autonomic sympathetic system, other systems are also activated: the sympatho-adrenal, renin–angiotensin–aldosterone, and hypothalamo–pituitary systems [50,51]. The features of sympathetic baroreflex and adaptive pressor systems functions are the main components of the orthostatic circulatory regulation system, which determine the phenotype of orthostatic hemodynamic changes. According to earlier studies, a stepwise increase in tilt angle during HUTT is accompanied by progressive neurohormonal shift with activation of all pressor systems [50]. Thus, at the same angle of inclination, taller subjects would have a higher hydrostatic column height, and the maximum difference in this indicator between tall and short subjects is observed in the upright position (90°). This means that in the upright position, orthostatic changes in hemodynamics depend on both the peculiarities of orthostatic regulation of blood circulation and on the subjects’ height, which determines the individual height of the hydrostatic column. This is the reason why it was necessary to level out the differences in height of the subjects to evaluate orthostatic regulation of blood circulation. Standard gravity load was provided by the individual tilt angle during HUTT (protocol Luanda) [29].

It should be considered that a person is exposed to an elevated hydrostatic pressure for about 16 h a day throughout one’s life. In the upright position, adaptive pressor systems are constantly activated (see above). A side effect of the activation of adaptive systems is the increase in vascular smooth muscle tone and transient increase of vascular wall stiffness.

The association of preclinical orthostatic abnormalities with early increases in vascular stiffness in healthy subjects defines the need to study their age-related evolution.

## 5. Limitations

A limitation of our study is the relatively small sample, which required careful interpretation of the results. This limitation was partly due to careful selection, especially in the older age groups to minimize the influence of comorbid status.

## 6. Conclusions

Tilt up is associated with a reversible adaptive increase in arterial stiffness.

The prevalence of orthostatic normotension in healthy adults reduces with age, it transforms into either preclinical orthostatic hypotension or hypertension.

Both preclinical orthostatic abnormalities are associated with higher baseline vascular stiffness (pulse wave velocity).

## 7. What Is New?

We used a HUTT standardized by hydrostatic column height (Luanda protocol) with concurrent PWV measurement to diagnose preclinical orthostatic abnormalities and analyze their association with vascular stiffness. The Luanda protocol provides a standard gravity load for all subjects regardless of height. Preclinical OH and OHT terminology was applied to emphasize the possible evolution of preclinical abnormalities into clinical orthostatic disorders.

## 8. Perspectives

A prospective cohort study of a healthy young population is needed to study the evolution of preclinical orthostatic abnormalities into clinical disorders. Future prospective studies will automate and personalize the assessment of arterial hypertension predictive risk-based approach and analyze individual risk according to a combination of various intrinsic and extrinsic factors, including lifestyle changes and other preventive measures [52].

## Figures and Tables

**Table 1:** Characterization of different age groups of subjects (*N* = 120).

Characteristics	Group 1 *n *= 40	Group 2 *n *= 40	Group 3 *n *= 40	Inter-Group Differences
Age Range, Years	20–30	31–45	Over 45	
Age, years	22 [20; 24]	37 [33; 38]	55 [49; 59]	*p*_1–2_ = 0.0002 *p*_1–3_ = 0.0007 *p*_2–3_ = 0.001 KW test: H = 105.9, *p* < 0.0001
Sex, male/female	18/22	17/23	19/21	
Height, cm	171.5 [157; 179]	173.5 [164; 182]	165 [158; 177]	*p*_1–2_ = 0.06 *p*_1–3_ = 0.017 *p*_2–3_ = 0.027 KW test: H = 9.67, *p* = 0.008
Weight, kg	65 [55; 79]	77.5 [63; 87.5]	75 [68; 85.5]	*p*_1–2_ = 0.076 *p*_1–3_ = 0.045 *p*_2–3_ = 0.07 KW test: H = 7.31, *p* = 0.026
Body mass index, kg/m^2^	22.5 [20.2; 24.1]	24 [22.5; 28.1]	26.1 [23.6; 30.6]	*p*_1–2_ = 0.026 *p*_1–3_ = 0.001 *p*_2–3_ = 0.23 KW test: H = 19.6, *p* = 0.0001

Note: The data are presented as Me [range]—median—for parameters that were not normally distributed, quartiles—[25%; 75%]—margins of the interquartile range, *p*—the significance of inter-group differences in selected characteristics. KW test—Kruskal–Wallis test.

**Table 2:** Health status of participants in different age groups.

Health Status	Group 1 *n *= 40	Group 2 *n *= 40	Group 3 *n *= 40
Healthy	37/92.5%	27/67.5%	22/55%
Arterial hypertension		6/15%	12/30%
Atherosclerosis		4/10%	6/15%
History of transient ischemic attack		1/2.5%	
Myopia	2/5%		
Chronic pancreatitis	1/2.5%		
Psoriasis		2/5%	
Bronchial asthma		2/5%	
Chronic bronchitis		1/2.5%	
Venous thrombosis		2/5%	
Gastritis		2/5%	
Diabetes mellitus		1/2.5%	5/12.5%
Uterine myoma			1/2.5%

Note: The data are presented as the number of separate nosologies in 3 age groups and as a percentage of the number of subjects in the group.

**Table 3:** Inter-group differences and intra-group orthostatic changes in BP and PWV indices.

Parameters Age Range, Years	Group 1	Group 2	Group 3	Inter-Group Differences			KW
	*n *= 40 20–30	*n *= 40 31–45	*n *= 40 >45	*p*_1–2_	*p*_1–3_	*p*_2–3_	
I Supine							
HR b/min	62 [57; 67.5]	68 [62.5; 77.5]	64.5 [58; 72]	**0.004**	0.39	0.29	1
SBP mmHg	120.3 [109.7; 126]	125.1 [119; 130.6]	125.6 [115; 136.6]	0.05	0.1	0.058	2
DBP mmHg	72.7 [66.2; 75.7]	81.5 [73.6; 86.8]	82.5 [73; 89.8]	**<0.001**	**0.001**	0.054	3
baPWV m/s	8.9 [8.3; 9.5]	10.1 [9.7; 11]	11.3 [10.7; 11.9]	**0.001**	**<0.001**	**<0.001**	4
cfPWV m/s	5.2 [4.7; 5.7]	6.3 [6; 6.9]	7.3 [6.6; 7.7]	**0.006**	**<0.001**	**0.002**	5
II HUTT							
HR b/min	74 [68.5; 78]	73 [69.5; 85]	67.5 [62.5; 75]	0.059	0.063	**0.015**	6
SBP mmHg	123.1 [108.5; 128.5]	126.3 [113.2; 134.5]	120.2 [112.8; 131.1]	0.25	0.061	0.059	7
DBP mmHg	79.9 [74; 82.5]	85.9 [79.4; 90.7]	84.3 [77.5; 90.8]	**0.003**	0.051	0.09	8
baPWV m/s	12.9 [11.9; 14.2]	13.8 [13; 14.5]	14.3 [13.3; 15.4]	0.16	**0.001**	0.26	9
III Return to supine							
HR b/min	62 [56; 66]	66 [59; 73.5]	63 [57.5; 68.5]	0.024	0.77	0.39	10
SBP mmHg	119.2 [109.7; 127.2]	126.2 [121; 134.5]	126.2 [118; 134.5]	**0.037**	0.08	0.09	11
DBP mmHg	74 [70.1; 78.5]	81 [73; 85.7]	79.7 [73; 85.2]	**0.002**	**0.02**	0.082	12
baPWV m/s	8.8 [8.4; 9.5]	10.3 [9.4; 10.8]	10.5 [9.7; 11]	**0.001**	**0.001**	0.097	13
Intra-group differences in orthostatic changes							
HR	***p_I–II_* = 0.001** *p_I–III_* = 0.14	***p_I–II_* = 0.001** ***p_I–III_* = 0.05**	***p_I–II_* = 0.001** *p_I–III_* = 0.051				
SBP	***p_I–II_* = 0.03** ***p_I–III_***= 0.06	*p_I–II_* = 0.93 *p_I–III_* = 0.91	*p_I–II_* = 0.27 *p_I–III_* = 0.51				
DBP	***p_I–II_* = 0.001** ***p_I–III_*** =0.055	***p_I–II_* = 0.001** *p_I–III_* = 0.63	***p_I–II_* = 0.004** *p_I–III_* = 0.15				
baPWV	***p_I–II_* = 0.001** *p_I–III_* = 0.97	***p_I–II_* = 0.001** *p_I–III_* = 0.6	***p_I–II_* = 0.001** ***p_I–III_* = 0.001**				

The data are presented in the form of Me [range]—median—for parameters that were not distributed normally, quartiles—[25%; 75%]—25–75% boundaries of the interquartile range. KW—Kruskal–Wallis test: 1—H = 10.01, *p* = 0.0067; 2—H = 6.91, *p* = 0.03; 3—H = 25.7, *p* < 0.0001; 4—H = 66.7, *p* < 0.0001; 5—H = 64.82, *p* < 0.0001; 6—H = 8.97, *p* = 0.011; 7—H = 3.0, *p* = 0.22; 8—H = 11.4, *p* = 0.003; 9—H = 13.32, *p* = 0.001; 10—H = 7.05, *p* = 0.029; 11—H = 7.51, *p* = 0.02; 12—H = 13.04, *p* = 0.001; 13—H = 33.5, *p* < 0.001. SBP—systolic blood pressure, DBP—diastolic blood pressure, HR—heart rate, baPWV—the brachial–ankle pulse wave velocity, cfPWV—carotid–femoral pulse wave velocity, inter-age group differences of these parameters are presented at the upper part of the table for each of the three positions: I—supine, II—HUTT, III—return to supine position (right side of the table). The significance of orthostatic changes in the selected parameters in the separated age groups is shown at the bottom of the table. *p_I–II_* depicts significance of the differences between parameters in supine and HUTT positions, *p_I–III_* denotes significance of the differences between the supine and return to supine positions, while *p_II–III_* represents significance of the differences between the HUTT and return to supine positions in each of the 3 age groups. Digits in bold mark statistically significant differences.

**Table 4:** Orthostatic changes in BP and PWV in individuals with and without preclinical orthostatic circulatory abnormalities.

	Group 1 Preclinical OH	Group 2 ONT	Group 3 Preclinical OHT	Inter-Group Differences			KW
	*n* = 20 (19.6%) ΔSBP < −5 mmHg	*n* = 64 (62.8%) ΔSBP ±5 mmHg	*n* = 18 (17.6%) ΔSBP > +5 mmHg	*p*_1–2_	*p*_1–3_	*p*_2–3_	
I. Supine							
HR b/min	66 [59; 76]	64 [57; 70]	67 [61; 72]	0.34	0.25	0.91	1
SBP mmHg	126.3 [121; 130.0]	119.9 [111.5; 126.2]	119.3 [110.3; 125]	**0.018**	**0.018**	0.87	2
DBP mmHg	85.5 [71.3; 90.6]	73.5 [76.7; 798]	76.6 [71.3; 82]	**0.001**	**0.02**	0.17	3
baPWV m/s	11.0 [10; 11.8]	9.5 [8.7; 10.4]	10.4 [10; 10.8]	**0.001**	0.12	**0.002**	4
cfPWV m/s	7.2 [6.3; 7.6]	5.9 [5.1; 6.6]	6.5 [6.1; 6.8]	**0.001**	0.15	**0.005**	5
II. HUTT							
HR b/min	70 [66; 79]	73 [68; 77]	72 [66; 82]	0.41	0.77	0.69	6
SBP mmHg	115.6 [112; 121.4]	119 [109; 128.3]	127.5 [123; 137]	0.31	**0.001**	**0.004**	7
DBP mmHg	82.1 [76.3; 86.3]	80 [75; 85.7]	86 [80.3; 91]	0.34	**0.014**	**0.012**	8
baPWV m/s	14.1 [13.3; 15.3]	13.2 [12.1; 14.4]	14.1 [13.1; 14.2]	**0.008**	0.06	**0.039**	9
III. Return to supine							
HR b/min	59 [58; 74]	63 [56; 69]	64 [61; 67]	0.53	0.32	0.67	10
SBP mmHg	126.5 [121; 134]	121.1 [109.4; 128.3]	126.7 [123; 138]	0.058	0.69	**0.045**	11
DBP mmHg	81 [75.5; 85.7]	76 [70; 80.8]	81 [72.5; 85.5]	**0.012**	0.78	**0.035**	12
baPWV m/s	10.6 [10.1; 11.7]	9.3 [8.5; 10]	10.3 [9.1; 10.6]	**0.006**	0.23	**0.021**	13
	Intra-group differences in orthostatic changes						
HR	***p_I–II_* = 0.01** *p_I–III_* = 0.81	***p_I–II_* < ** **0.001** ***p_I–III_* < ** **0.006**	***p_I–II_* < 0.001** *p_I–III_* = 0.064				
SBP	***p_I–II_* = 0.001** *p_I–III_* = 0.9	*p_I–II_* = 0.48 *p_I–III_* = 0.048	***p_I–II_* = ** **0.001** ***p_I–III_* < ** **0.001**				
DBP	*p_I–II_ **=*****0.001** *p_I–III_* = 0.5	***p_I–II_* = 0.001** *p_I–III_* = 0.17	***p_I–II_* = 0.001** *p_I–III_* = 0.21				
baPWV	***p_I–II_* = 0.001** *p_I–III_* = 0.84	***p_I–II_* = 0.001** *p_I–III_* = 0.39	***p_I–II_* = 0.001** *p_I–III_* = 0.18				

Note: the data are presented in the form of Me [range]—median—for parameters that were not distributed normally, quartiles—[25%; 75%]—25–75% boundaries of the interquartile range. KW—Kruskal–Wallis test: 1—H = 1.68, *p* = 0.43; 2—H = 12.46, *p* = 0.002; 3—H = 17.38, *p* = 0.0002; 4—H = 20.1, *p* < 0.0001; 5—H = 20.21, *p* < 0.0001; 6—H = 0.37, *p* = 0.82; 7—H = 12.51, *p* = 0.002; 8—H = 10.01, *p* = 0.007; 9—H = 11.08, *p* = 0.0039; 10—H = 1.34, *p* = 0.51; 11—H = 8.31, *p* = 0.016; 12—H = 8.57, *p* = 0.014; 13—H = 16.6, *p* < 0.0003. SBP—systolic blood pressure, DBP—diastolic blood pressure, HR—heart rate, baPWV—brachial–ankle pulse wave velocity, cfPWV—carotid–femoral pulse wave velocity (only baseline values) differences of these parameters in groups of subjects with and without subclinical orthostatic abnormalities are presented in Table 4 for each of the three positions: I—supine, II—HUTT, III—return to supine position, inter-group differences—in the right side of the table. The significance of orthostatic changes in the selected parameters in the separated groups are shown at the bottom of the table. *p_I–II_* depicts significance of the differences between parameters in supine and HUTT positions, *p_I–III_* denotes significance of the differences between the supine and return to supine positions, while *p*_II–III_ represents significance of the differences between the HUTT and return to supine positions in each of the 3 groups. Digits in bold mark statistically significant differences.

## Data Availability

Not applicable.
